# Allelopathic effects of glucosinolate breakdown products in Hanza [*Boscia senegalensis* (Pers.) Lam.] processing waste water

**DOI:** 10.3389/fpls.2015.00532

**Published:** 2015-07-14

**Authors:** Loren J. Rivera-Vega, Sebastian Krosse, Rob M. de Graaf, Josef Garvi, Renate D. Garvi-Bode, Nicole M. van Dam

**Affiliations:** ^1^Department of Entomology, Pennsylvania State University, University Park, PAUSA; ^2^Molecular Interaction Ecology, German Centre for Integrative Biodiversity Research (iDiv) Halle-Jena-Leipzig, LeipzigGermany; ^3^B-WARE Research Centre, NijmegenNetherlands; ^4^Molecular Interaction Ecology, Institute of Water and Wetland Research, Radboud University, NijmegenNetherlands; ^5^Ecological Microbiology, Institute of Water and Wetland Research, Radboud University, NijmegenNetherlands; ^6^Sahara Sahel Foods, ZinderNiger Republic; ^7^Institute of Ecology, Friedrich Schiller University Jena, JenaGermany

**Keywords:** Africa, allelopathy, ecosystem services, famine food, glucocapparin, methylisothiocyanate, weed control

## Abstract

*Boscia senegalensis* is a drought resistant shrub whose seeds are used in West Africa as food. However, the seeds, or hanza, taste bitter which can be cured by soaking them in water for 4–7 days. The waste water resulting from the processing takes up the bitter taste, which makes it unsuitable for consumption. When used for irrigation, allelopathic effects were observed. Glucosinolates and their breakdown products are the potential causes for both the bitter taste and the allelopathic effects. The objectives of this study are to identify and quantify the glucosinolates present in processed and unprocessed hanza as well as different organs of *B. senegalensis*, to analyze the chemical composition of the processing water, and to pinpoint the causal agent for the allelopathic properties of the waste water. Hanza (seeds without testa), leaves, branches, unripe, and ripe fruits were collected in three populations and subjected to glucosinolate analyses. Methylglucosinolates (MeGSL) were identified in all plant parts and populations, with the highest concentrations being found in the hanza. The levels of MeGSLs in the hanza reduced significantly during the soaking process. Waste water was collected for 6 days and contained large amounts of macro- and micronutrients, MeGSL as well as methylisothiocyanate (MeITC), resulting from the conversion of glucosinolates. Waste water from days 1–3 (High) and 4–6 (Low) was pooled and used to water seeds from 11 different crops to weeds. The High treatment significantly delayed or reduced germination of all the plant species tested. Using similar levels of MeITC as detected in the waste water, we found that germination of a subset of the plant species was inhibited equally to the waste water treatments. This confirmed that the levels of methylisiothiocyanate in the waste water were sufficient to cause the allelopathic effect. This leads to the possibility of using hanza waste water in weed control programs.

## Introduction

**FIGURE 1:**
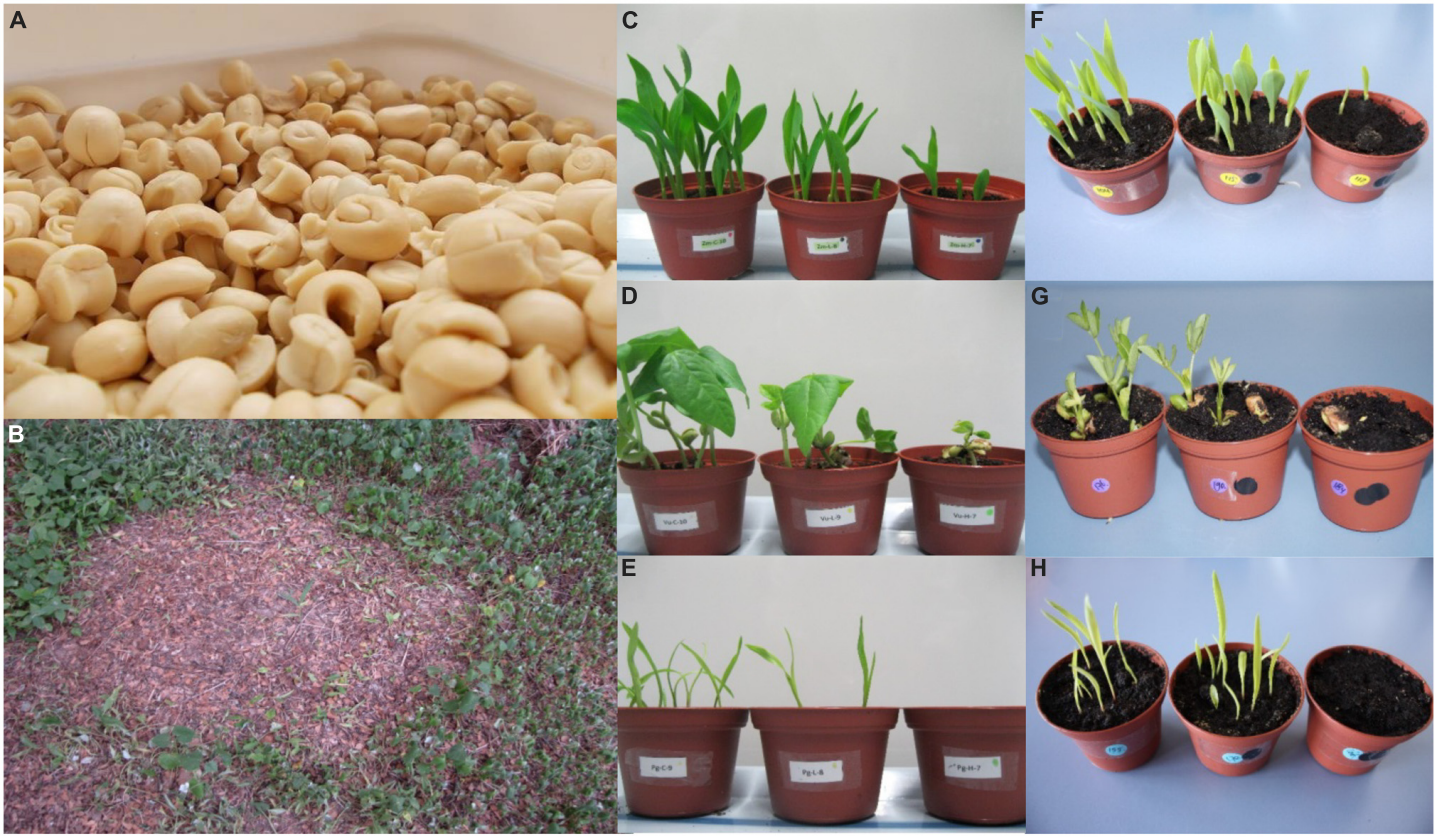
**(A)**: Hanza, **(B)** Effect of watering standing vegetation with hanza waste water in Zinder, Niger. **(C–E)**: effects of hanza waste water on germination of *Zea mays* **(C)**, *Vigna unguiculata* **(D)**, and *Pennisetum glaucum* **(E)**. Treatments (from left to right in each picture): Tap water, Low (waste water days 4–6), and High (days 1–3); **(F–H)**: effect of methylisothiocyanate (MeITC) on seed germinations of *Z. mays* **(F)**, *Arachis hypogaea* **(G)**, and *P. glaucum* **(H)**. Treatments (from left to right in each picture): Tap water, 0.2 mM MeITC, 1.0 mM MeITC. Picture credits: Renate Garvi **(A,B)**, Loren J. Rivera-Vega **(C–E)**, Nicole M. van Dam **(F–H)**.

During times of famine in Africa the seeds of the drought resistant native evergreen shrub, *Boscia senegalensis*, locally known as hanza or mukheit, among others, are often used as food in sub-Saharan countries (Orwa et al., 2009). In Niger, the trees flower in October–November and the fruits take until summer of the next year (June–August) to fully ripen. The ripe fruits contain a sweet jelly that can be consumed directly or used to produce syrup, and 1–4 seeds [Agroforestry Database 4.0 (Orwa et al., 2009)]. After removal of the carpels and testa (seed coats) of the seeds, the remaining cotyledons plus the embryo, hereafter referred to as “hanza”, are used for consumption as well (**Figure 1A**). Hanza is a rich source of starch (40–66% of dry mass), protein (15–30% of dry mass), and minerals (K, P, Si, and Mg; Booth and Wickens, 1988; Salih et al., 1991). The seeds can be consumed after soaking or cooking in hot water, or used to produce flour that can be used for baking and porridge (Booth and Wickens, 1988). However, *B. senegalensis* belongs to the Capparaceae family and is known to contain glucosinolates (Kjær et al., 1973). Glucosinolates are nitrogen and sulfur containing secondary metabolites present in species belonging to the order Brassicales. The breakdown products of glucosinolates, such as isothiocyanates, are formed upon contact with the plant-produced enzyme myrosinase that is stored in separate cells in the plant (Halkier and Gershenzon, 2006; Hopkins et al., 2009). It was found that leaves and fruits of *B. senegalensis* indeed produce isothiocyanates upon crushing and consequently they can be effectively used to protect grains from storage pests (Seck et al., 1993; Gueye et al., 2011, 2013a). Even though it has been shown that *B. senegalensis* leaves and whole fruits contain MeGSL, also known as glucocapparin (Gueye et al., 2011, 2013a,b), the glucosinolate levels in hanza itself were never explicitly assessed.

Consumers have developed a process to eliminate the toxic and bitter compounds from hanza. The procedure consists of soaking the seeds in water for 4–7 days while changing the water daily (Salih et al., 1991; Garvi, personal communication). Although this process allows for the use of hanza for nutrition, it also requires large amounts of clean water, a limiting resource in these countries. The region suffers from what is considered economic water scarcity, where the infrastructure and investments in water are not enough to meet population demands (IFPRI, 2013; UN, 2014). The water takes up the bitter taste of the hanza seeds which limits uses for human consumption or husbandry. Therefore, identifying alternative uses for this waste water is of high relevance. One of the initial alternatives considered using *B. senegalensis* waste water for irrigation of standing crops. However, it was observed that plants watered with the waste water wilted and in some cases died, thus showing allelopathic properties (**Figure 1B**). Given the scarcity of water in the region, this reduces the potential to re-use the waste water for crop irrigation. Moreover, for purposes of water recycling (purification and re-use) it has to be known which contaminants are in the water that should be removed after the production of sweet hanza to make the water suitable for re-use. Given the fact that crushed leaves of hanza produce methylisothiocyanate (MeITC; Gueye et al., 2013a) and that MeITC has strong allelopathic properties (Brown and Morra, 1997), it is likely that the glucosinolates in the hanza are converted into MeITC during the processing and that this causes the observed allelopathic effect.

To test our assumptions, we analyzed the levels of glucosinolates and its breakdown products in hanza and the waste water resulting from hanza processing. Specifically, we quantified the concentrations of glucosinolates in *B. senegalensis* leaves, fruits and hanza (i.e., the seeds without testa) collected in different years and populations in Niger. Moreover, we analyzed the concentration of glucosinolates in hanza during the debittering process and chemically analyzed the waste water for glucosinolates and their breakdown products as well as for macro- and micro elements. Based on these results, we separately tested the effect of waste water collected after the first 3 days of processing and after 4–6 days of processing on the germination of 11 different crop and weed species. A sub-set of these species was used to confirm that indeed the concentrations of MeITC we found in the waste water were sufficient to cause the observed effects. Based on our results, we conclude that hanza contains very high levels of MeGSL, which significantly decrease during the soaking process. The resulting waste water contains MeITC in concentrations that are sufficient to inhibit or delay seed germination. We discuss if and how it is possible to apply hanza waste water as a way to apply weed control.

## Materials and Methods

### Origin of Reference Compounds

Sinigrin, MeITC (97% pure) and ethylisothiocyanate (97% pure) were all purchased at Sigma–Aldrich (St Louis, MA, USA). A reference sample of MeGSL (glucocapparin) was kindly provided by Dr. Jacqueline Bede, McGill University, Canada.

### Sampling of Hanza Batches and Organs of *Boscia senegalensis* for Glucosinolate Concentrations

Hanza (*B. senegalensis* seeds without the testa) and various organs of *B. senegalensis* shrubs were sampled in different years and in different populations. In 2012, we analyzed five batches of dry, unprocessed hanza harvested in 2011 at different locations and dates in the Zinder Region of Niger (Atalouwawa, collection date October 07, 2011, Tirmini; September 16, 2011, El Gada: October 17, 2011; Aroungouza: July 10, 2011, Tanout: June 26, 2011).

In 2013, old leaves, young leaves, branches, unripe fruits, and seeds (hanza) were collected from three different populations (Baboul, Tanout, and Zinder), air dried and shipped to Nijmegen, NL. Branches could not be reliably analyzed because they were too tough to be ground to a fine powder either by the ball mill or a shredder. Additionally, fresh fruits from five individual trees in the Zinder population collected in September 2013 were bagged and taken to Nijmegen for analysis within 40 days. Directly after arrival, the fruits were separated in three parts: the outer carpel, the inner carpel (including testa) and embryo plus cotyledons (hanza), and analyzed following standard procedures. One of the batches showed significant mold development and was not included in the chemical analyses. In 2014, another batch of dry unprocessed hanza from the Zinder population (population Kanya Wamé, Zinder, Niger) was sent to Leipzig, Germany. This batch was used to produce waste water for the germination tests.

Differences in glucosinolate concentrations between batches within 1 year were determined using ANOVA or MANOVA analyses (STATISTICA vs. 10.0, Statsoft, Tusla, OK, USA). Levene’s test was used to check for the assumption of HOV, and analyses of the residuals were used to check for assumptions of normality.

### Effect of Soaking Hanza on Seed Glucosinolate Content and ITC in Water

Six 50 ml tubes were filled with 5 g of dried seeds (random mix of hanza populations collected in 2011, **Table 1**) each and 40 ml tap water (8x seed volume). The tubes were placed in a climate chamber at 35°C, 50% R.H., 12hL/12hD. Each day the water was decanted, collected and replaced with fresh tap water. The pH of the decanted waste water was measured each day to establish a potential correlation between the level of glucosinolates in the seeds and the pH of the waste water. Both seeds and water samples were frozen at -20°C until analysis. After 2 days, the amount of water was reduced to 20 ml (4x seed volume). The seeds were soaked for 7 days in total.

**Table 1:** Average concentration (μmoles.g^-1^ dry mass; SEM between brackets) of methylglucosinolate (MeGSL) in different organs of *Boscia sengalensis* in different years and populations in Niger, Africa.

Year 2011					
*Hanza batches*					
**Population**	***n***	**Hanza**

Atalouwawa	3	96.6 (4.1)^ab^
Tirmini	3	89.1 (2.3)^b^		
El Gada	3	91.5 (1.6)^ab^		
Aroungouza	3	89.3 (3.2)^b^		
Tanout	3	103.3 (1.9)^a^		

**Year 2013**					
***Individual seeds (Hanza), old and young leaves***					

**Population**	***n***	**Hanza**	**Unripe fruits**	**Old leaves**	**Young leaves**

Baboul	5	197.9 (13.9)	72.8 (5.6)	59.2 (6.8)	65.9 (6.6)
Tanout	5	261.9 (24.2)	71.4 (6.6)	73.5 (12.1)	59.3 (11.1)
Zinder	5	230.4 (29.2)	67.4 (9.9)	60.3 (16.7)	102.7 (17.7)

***Ripe fruits (individual)***					

**Population**	***n***	**Hanza**	**Inner carpel**	**Outer carpel**	

Zinder	4	113.1 (26.4)^a^	1.3 (0.8)^b^	6.2 (3.0)^b^	

**Year 2014**					
***Hanza seed batch***					

**Population**	***n***	**Hanza**			

Kanya Wamé, Zinder	3	230.7 (20.5)			

*Different letters indicate significant differences in concentrations between hanza within 1 year (2011, Tukey *Post hoc* HSD, *P* < 0.05) or between parts of unripe fruits (Friedman ANOVA, followed by multiple comparisons *Z*-tests, *P* < 0.05).*

### Glucosinolate Analysis

The seed samples taken from the tubes as well as from the original seed batch were freeze-dried and ground in a Retsch ball mill. Aliquots (*n* = 3 per day) of 50 mg were weighed into a 2 ml Eppendorf tube and extracted following standard procedures (EC, 1990). The resulting desulfo-glucosinolates were analyzed on an HPLC (Ultimate 3000, Dionex, Idstein, Germany) with PDA-detector equipped with a C18 column (150 mm × 4.6 mm i.d., 5 μm particle size) plus pre-column (Alltima, Grace Davison Discovery Sciences, Lokeren, Belgium). The flow was maintained constant at 0.750 ml/min, with a gradient starting from 2% acetonitrile in water to 20% acetonitrile after 15 min. The column was kept at 40°C and the sample injection volume was 10 μl. Detection of the peaks with the PDA detector was at 229 nm with a reference wavelength of 600 nm. The identity of desulfo-MeGSL (Rt = 3.5 on the above HPLC system) was confirmed using a reference sample kindly provided by Dr J. Bede, and on LC-MS (Supplementary Figure S1). An external sinigrin curve (50–650 μM) was used for quantification; the response factor was set to 1.0 (Brown et al., 2003).

### ITC Analysis of Waste Water

Isothiocyanates were extracted by using 800 μl of the above waste water samples. To verify the quality of the extraction to each sample 400 μl of a 1.74 mM ethylisothiocyanate solution in milliQ water was added as internal standard. To extract the isothiocyanates from the water, 200 μl of dichloromethane (DCM) was added, the mixed sample was vortexed, briefly centrifuged at 10000 rpm and 100 μl of the DCM phase was taken for GC-TOF-MS analyses on a JEOL AccuTOF-GCv JMS-100GCv equipped with an Agilent 7890A GC with a HP-5MS column (30 m × 0.25 mm × 0.25 μm) and a G4513A autosampler. Conditions used for the GC-TOF-MS isothiocyanate analyses: 50°C for 1 min, followed by a temperature gradient of 30°C/min to 200°C. Split ratio: 1:10. Detector voltage 2000 V. Injection volume 1 μl. An external MeITC reference curve (5–20 nmol/microliter) was used for quantification.

### Seed Sources

Seeds of the following plant species (common names in brackets) were obtained from the following sources: *Brassica nigra* (Black mustard) – personal collection NM van Dam, population “Proefveld Wageningen,” The Netherlands, 2009; *Brassica juncea* cv. varuna (Brown mustard) – Division of Genetics, IARI, New Delhi, India (see Mathur et al., 2013); *Zea mays* (corn) “Zuckermays F1, Tasty Gold” and *Solanum lycopersicon* cv. “Hellfrucht Hilmar” (tomato) – Weigelt GmbH & Co, Walluf, Germany; *S. dulcamara* (bittersweet nightshade) – collection Solanaceae Genebank Radboud University Nijmegen, the Netherlands; *Lolium perenne* (perennial ryegrass)– Veevoeder- en Kunstmesthandel J.J. Lamers V.O.F., Heteren, The Netherlands. Seeds of West-African crops species such as *Vigna unguiculata* (local name ‘Niébé’ or cowpea), *Hibiscus sabdariffa* (local name ‘Oseille’), *Sorghum bicolor* (sorghum, abbreviated as *Sor. bicolor* to avoid confusion with *Solanum*), *Pennisetum glaucum* (millet), and *Arachis hypogea* (peanut) were all obtained at a local market in Zinder, Niger, by Renate Garvi and sent to Germany for germination assays.

### Hanza Water for Germination Experiments

Two batches of hanza waste water were produced for germination assays by soaking 400 g of hanza (population Kanya Wamé, Zinder, Niger) each time in tap water. Batches were kept at 30°C day and night inside a growth chamber. Water was collected daily and replaced with clean water. For days 1 and 2, seeds were soaked in 3.2 L of water, from day 3 onward the volume of water was cut in half (1.6 L). Waste water from days 1–3 and 4–6 were pooled and named “High” and “Low,” respectively. Seeds imbibed approximately 2 L of water during the first 3 days and 0.5 L during the last 3 days. Two sets of High and Low waste water pools were made in August and September 2014, respectively.

### Hanza Water Chemical Analyses

Two independent samples of the High and Low waste water pools produced in August 2014, and one High and Low sample of the September 2014 pools, plus three samples of the tap water used to soak the Hanza were taken for elemental analyses. Per sample, 10 ml supplemented with 0.1 ml 65% HNO_3_ to prevent metal precipitation, was analyzed using ICP-OES (Inductively coupled plasma – optical emission spectrometry; iCAP 6000 Thermo Fisher Scientific, Waltham, MA, USA) to assess the elemental composition of the water (Butcher, 2010). In addition, an 18 ml aliquot was used to assess total organic carbon and total organic nitrogen concentrations on a Total Organic Carbon/Nitrogen analyzer (TOC-L CPH/CPN analyzer, Shimadzu, Duisburg, Germany).

The same pools were used to assess the glucosinolate levels in the water, but only one sample was analyzed per time point and batch (*n* = 2). Per pool, 0.5 ml of waste water was brought directly on a DEAE A25 Sephadex column as if they were regular glucosinolate extracts and processed as such from there. Three samples of the seed batch used to produce the waste water were extracted as well to calculate the amount of MeGSL that was present in the seeds at the start of the soaking process. Differences in the chemical composition of the waste water batches and tap water were identified using MANOVA, followed by ANOVA, and Tukey *Post hoc* HSD analyses per element or compound using STATISTICA 10.0 software [StatSoft, Inc. (2011), Tusla, OK, USA].

### Germination Assay with Hanza Waste Water

For each plant species, 10 plastic pots (Teku, 7 cm ø, 200 ml volume) were filled with multiplication substrate (Floraton 3, Floragard, Oldenburg, Germany). Each pot was sowed with 10 or 5 (for *A. hypogaea*) seeds and lightly covered with a layer of ∼5 mm of multiplication mixture. Next, 50 mL of water from each treatment were used to water the pots. This completely saturated the soil in the pots. Treatments included: Control (tap water), High treatment (water pooled from days 1–3) and Low treatment (water pooled from days 4–6). The pots were covered with clear plastic household wrap, fixed with a rubber band around the pot, and placed in a climate cabinet set to 25°C daytime temperature and 22°C nighttime temperature, 70% R.H. and a 12 h photoperiod to mimic the conditions in Niger. To avoid cross contamination via the water, pots subjected to the same treatment were placed together on one plate (20 cm × 20 cm). Number of seeds germinated was counted 7 and 10 days after sowing to determine germinability. Above ground biomass was collected and air dried in an oven at 60°C for 24 h to measure dry weight. The dry biomass per pot was assessed by weighing to the nearest 0.1 mg. One-way ANOVA followed by a Tukey means of separation were used to assess significance.

### Germination Assay with MeITC Solutions

A subset of the above plant species, *Z. mays*, *B. nigra*, *S. lycopersicon*, *P. glaucum*, and *A. hypogaea*, was used to test the hypothesis that MeITC in the waste water was the causal agent for the effect of waste water on seed germination. MeITC (Sigma–Aldrich, 97% pure) was dissolved in warm tap water (Teasdale and Taylorson, 1986) to obtain a 1 mM (73 mg/L) and a 0.2 mM (14.7 mg/L) solution. Right after sowing, the pots were watered either with 50 ml tap water, 50 ml of the 1 mM (High) MeITC solution or 50 ml of the 0.2 mM (Low) MeITC solution. Per treatment level (Control, High, and Low) there were six plates, each containing one replicate pot per species (*n* = 6 pots per species per treatment). The 18 plates with the different treatments were evenly spread over the different positions in the cabinet using the same conditions as above, and the pots were randomly assigned to a position on the plate. The pots were checked daily for visible signs of germination. After 2 days the emerging *P. glaucum* seedlings in the control and Low treatments were touching the plastic, and the covers of all pots were removed. After removal of the covers each pot received another 5 ml of the respective treatment solution. From then on, the pots were bottom watered with tap water (80–100 ml per day). From 2 days after sowing, the number of seeds that had germinated, as evidenced by an emerging (hypo)cotyledon, epicotyledon, or coleoptyle in each pot was counted. Germination trajectories in the different treatments groups were analyzed using Kaplan–Meier time-to-event Survival Analyses (McNair et al., 2012) using STATISTICA 10.0 software.

## Results

### Methylglucosinolate is Present in all Tissues of *Boscia senegalensis*

Methylglucosinolate was found in all organs of *B.senegalensis* (**Table 1**). In contrast to a previous study (Kjær et al., 1973), we found no additional glucosinolates to be present. The levels of glucosinolates in hanza varied among populations and years. The five populations sampled in 2011, all showed concentrations around 100 μmol per gram dry mass (**Table 1**), whereby the hanza of the Tanout population had the highest concentration (ANOVA, *F*_4,10_ = 4.673, *P* = 0.0231). Overall, young and old leaves, whole unripe fruits and hanza showed significant differences between populations in 2013 (ANOVA, population effect, *F*_2,48_ = 4.42, *p* = 0.0173). As before, the average MeGSL concentrations were the highest in the Tanout population. MeGSL concentrations significantly differed between plant organs: the concentrations in the hanza itself were about 2 to 4 times higher (organ effect, *F*_3,48_ = 79.09, *p* < 0.001) than those in leaves or unripe fruits. This effect was consistent over all populations (population x organ effect, *F*_6,48_ = 1,10, *p* = 0.37). Interestingly, in ripe fruits, the MeGSL concentrations in inner and outer layers of the fruit wall (carpels) were extremely low, about 20 to 80 times lower than in the hanza (embryo plus cotyledons, Kruskal–Wallis-Test: *H* = 7.538, *p* = 0.0231), which may also explain the relatively low overall concentration in whole unripe fruits.

### Soaking Hanza Reduces Glucosinolates Levels and Produces Waste Water with Methylisothiocyanate

**FIGURE 2:**
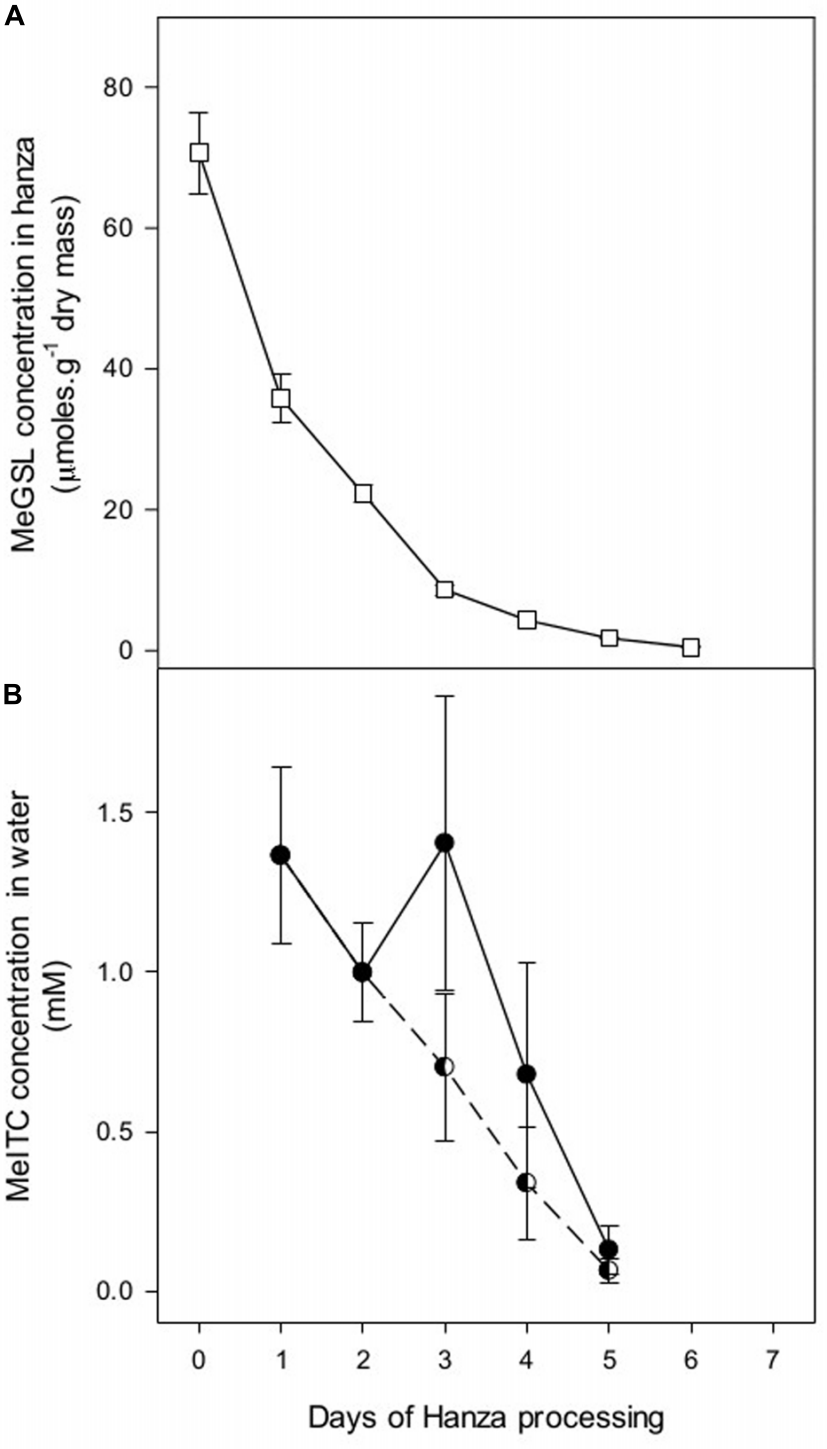
**(A)** Concentration of methylglucosinolate (MeGSL; +SEM) in hanza (mix of five populations collected in 2011) at different days after starting the soaking process. **(B)** MeITC (+SEM) concentration in waste water during the soaking process. The concentration of MeITC in the waste water collected after 6 days was below the detection limit. Half full circles connected by the dotted line indicate the theoretical MeITC concentrations when taking into account that from day 3 onward, the volume of fresh water to process hanza was reduced to 50% of that used on days 1 and 2.

Methylglucosinolate levels in hanza decreased with time to less than 10% of the original concentration within 5 days of soaking (**Figure 2A**). The strongest decrease occurred during the first 3 days of processing. The average pH value of the waste water collected on each day ranged from 6 to 6.7 and was not significantly correlated with glucosinolate levels in the hanza (data not shown). Waste water from the soaking process was analyzed to determine its content of MeITC – a conversion product of MeGSL (Supplementary Figure S1). When corrected for dilution effects after day 3, when the volume of water to soak the hanza was halved, the MeITC in waste water decreased at similar rates as MeGSL in seeds (**Figures 1C–E** and **2B**). This indicates that MeGSL is being converted to MeITC during the soaking process.

### Hanza Processing Waste Water has Allelopathic Properties

**FIGURE 3:**
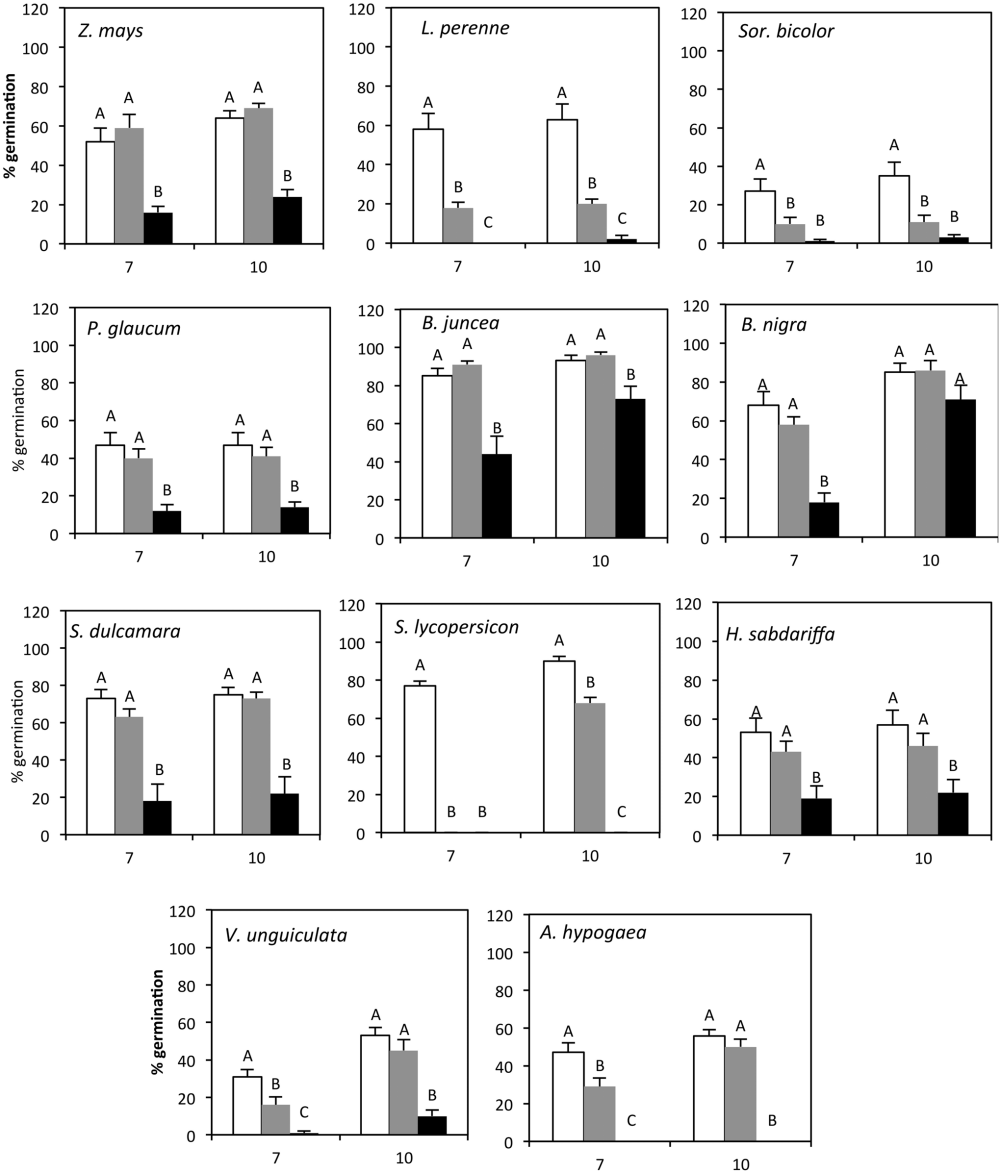
**Germination percentages (+SEM) at 7 and 10 days after sowing per treatment group: Control (white open bars), Low (gray bars), and High (black bars).** Different letters represent statistically significant differences in germination (One-way ANOVA, Tukey mean separation, *p* < 0.05).

**FIGURE 4:**
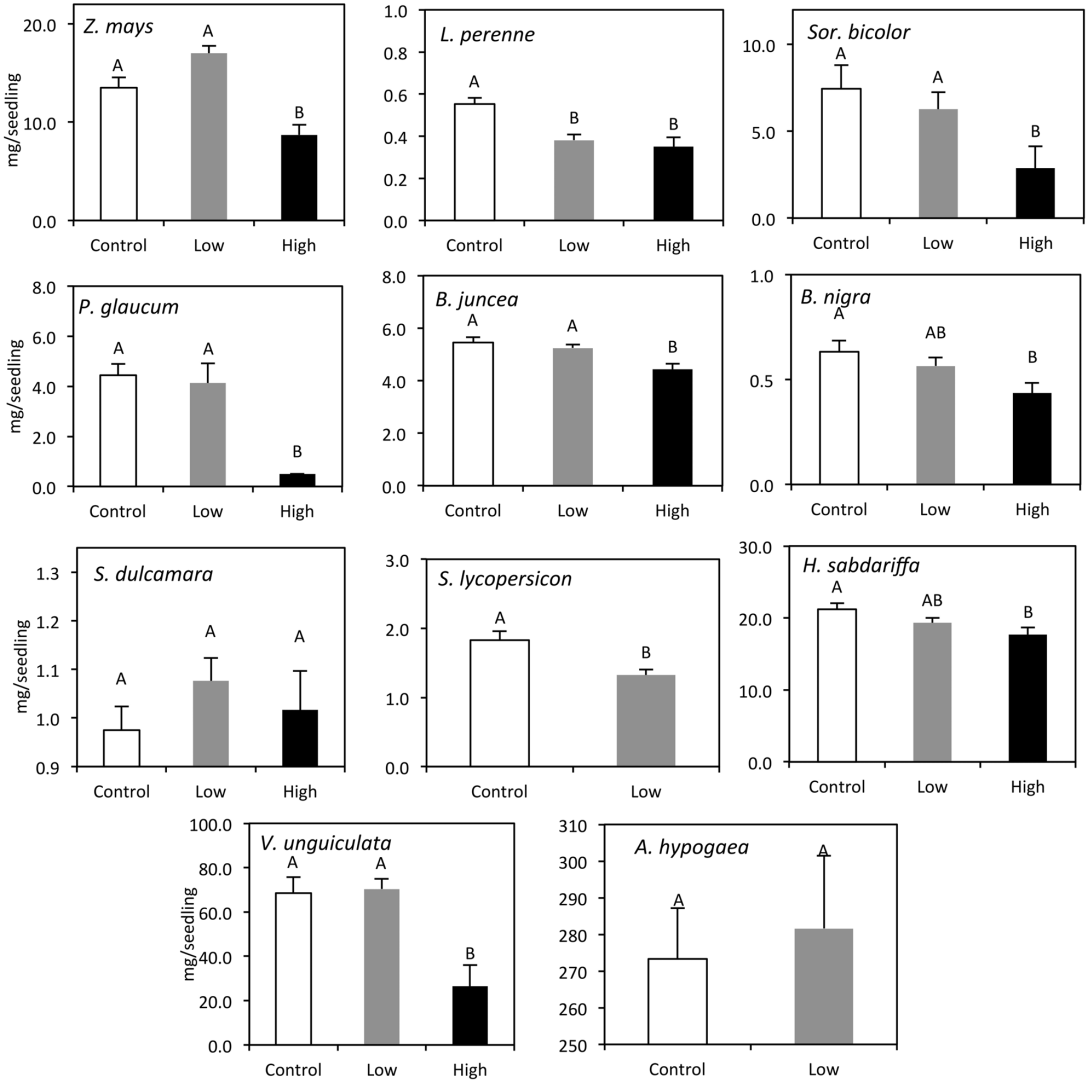
**Dry mass of seedlings (+SEM) after 14 days in each treatment group: Control (white open bars), Low (gray bars), and High (black bars).** Different letters represent statistically significant differences in dry weight (Tukey mean separation, *p* < 0.05).

After 7 days, watering seeds with waste water of the first 3 days (High treatment) had a significant negative effect on germination rates for all plant species tested (**Figure 3**). Hanza waste water of day 3–6 (Low treatment) had a significant effect (ANOVA, *p* < 0.05) on the germination of *L. perenne*, *Sor. bicolor*, *S. lycopersicon*, and *A. hypogaea*, only. After three more days of watering with clean water, the germination percentages in the waste water treatments had caught up. At that time, the Low treatment had a significant effect on *S. bicolor*, *P. glaucum*, and *S*. *lycopersicon* germination only. The High treatment still had a significant effect on most plant species tested except for *B. nigra* (**Figure 3**). After 10 days the dry weight of the seedlings was measured. The Low treatment had a significant effect on the biomass of *L. perenne*, *S. lycopersicon*, and *Sor. bicolor*. High treatment had a negative effect on the dry mass of all plant species, except for *S. dulcamara* (**Figure 4**). No seedlings from the High treatment were available to assess dry weight for *S. lycopersicon* and *A. hypogaea*.

### Chemical Composition of Waste Water Differs Significantly

Samples of water used for germination experiments were analyzed for elemental composition. In general, the waste water resulting from the debittering process has a high concentration of several macro and micro elements, with significant differences in quantity of basic elements among treatments (**Table 2**). The most abundant macro elements in both High and Low water were C, N, and S, showing that the soaking not only reduces the level of glucosinolates, but also the nutritive value of hanza. Water from the High treatment had the highest quantity of macro elements, except for Na, Ca, and Si, which were the highest in tap water. The most abundant micronutrients were B and Zn in all water samples: the amount of B in High water was almost five times as much as that in Low and tap water. Interestingly, a high concentration of Mn was found in High and Low water but not in tap water. Iron (Fe) levels were the highest in the low water indicating that this essential nutrient leaches out of the hanza with time.

**Table 2:** Elemental composition (macro elements, concentration in mM, micro elements: concentration in μM, MeGSL in mM) of tap water, waste water of days 1–3 (High) or of days 4–6 (Low) collected during the soaking of Hanza (*Boscia senegalensis* seeds).

	Tap water	High (days 1–3)	Low (days 4–6)	*F*_2,6_
**Macroelements (mM)**				
C	1.392 (0.288)^c^	332.861 (19.75)^a^	38.822 (5.466)^b^	706.5***
N	0.503 (0.041)^c^	41.907 (1.931)^a^	6.071 (0.525)^b^	1134.0***
P	n.d.	0.970 (0.048)^a^	0.276 (0.035)^b^	629.4***
S	1.663 (0.058)^b^	23.652 (2.762)^a^	3.772 (0.921)^b^	156.2***
K	0.096 (0.007)^b^	8.402 (0.338)^a^	0.592 (0.183)^b^	1323.8***
Mg	0.390 (0.026)^b^	1.330 (0.048)^a^	0.405 (0.165)^b^	86.1***
Na	2.196 (0.160)^a^	0.028 (0.011)^b^	0.395 (0.323)^b^	93.4***
Ca	1.200 (0.016)^a^	0.948 (0.005)^ab^	0.754 (0.215)^b^	9.7*
Si	0.201 (0.002)^a^	0.0128 (0.006)^b^	0.016 (0.012)^b^	667.3***
**Microelements (μM)**				
As	0.033 (0.032)	0.018 (0.031)	0.014 (0.018)	0.35^n.s.^
B	5.230 (0.207)^b^	24.073 (1.369)^a^	5.695 (2.757)^b^	109.2***
Cd	0.003 (0.003)	0.006 (0.001)	0.002 (0.002)	2.67^n.s.^
Co	<0.001^b^	0.015 (0.008)^a^	0.004 (0.006)^ab^	5.4*
Cu	0.761 (0.003)^b^	2.946 (0.084)^a^	0.163 (0.100)^c^	1138.4***
Fe	0.145 (0.057)	1.063 (0.198)	6.851 (4.736)	5.3*
Hg	0.011 (0.004)^a^	0.002 (0.002)^b^	0.005 (0.002)^ab^	7.4*
Mn	0.036 (0.004)^b^	13.544 (0.690)^a^	11.869 (3.168)^a^	46.4***
Mo	0.009 (0.006)^b^	0.062 (0.009)^a^	0.011 (0.003)^b^	72.3***
Ni	1.299 (0.023)^a^	1.147 (0.074)^b^	0.269 (0.023)^c^	426.1***
Pb	0.031 (0.026)	<0.001	<0.001	4.1^n.s.^
Sr	1.544 (0.028)^a^	0.079 (0.001)^c^	0.131 (0.092)^b^	677.7***
Zn	14.733 (0.813)^ab^	8.126 (2.629)^b^	20.175 (7.525)^a^	5.1*^p^* = ^0.051^
MeGSL (mM)		7.11 (0.98)	0.41 (0.05)	

N = 3, SD between brackets. MANOVA to test for differences between water samples over all elements: *F*_*12,2*_ = 119.206, *P* = 0.008. Last column: *F*-values of ANOVA per element. Significance: n.s. = not significant, *p < 0.05, **p < 0.01, ***p < 0.001.Different letters indicate significant differences between water samples based on Tukey HSD *post hoc* analyses.

Furthermore we found high levels of MeGSL in the high waste water (**Table 2**). Based on how much water was used (8 L for high, 4.8 L for low), estimates of how much was lost during soaking due to absorption by the dry hanza and evaporation (2 L and 0.5 L in high and low, respectively) and the initial levels of MeGSL in hanza (**Table 1**, 2014 batch), we calculated that of the 92.3 mmol of MeGSL present in 400 g seeds, 42.2 mmol, or ∼45%, is retrieved in waste water of the first 3 days, and 1.9 mmol (∼2%) in the waste water of the last 3 days. Seen the very low levels of glucosinolates that remain in the seeds, this means that more than 50% of the MeGSL is lost during soaking, probably because of conversion into MeITC followed by evaporation or possibly because other breakdown products are formed.

### Methylisothiocyanate in the Waste Water is Responsible for Germination Inhibition

**FIGURE 5:**
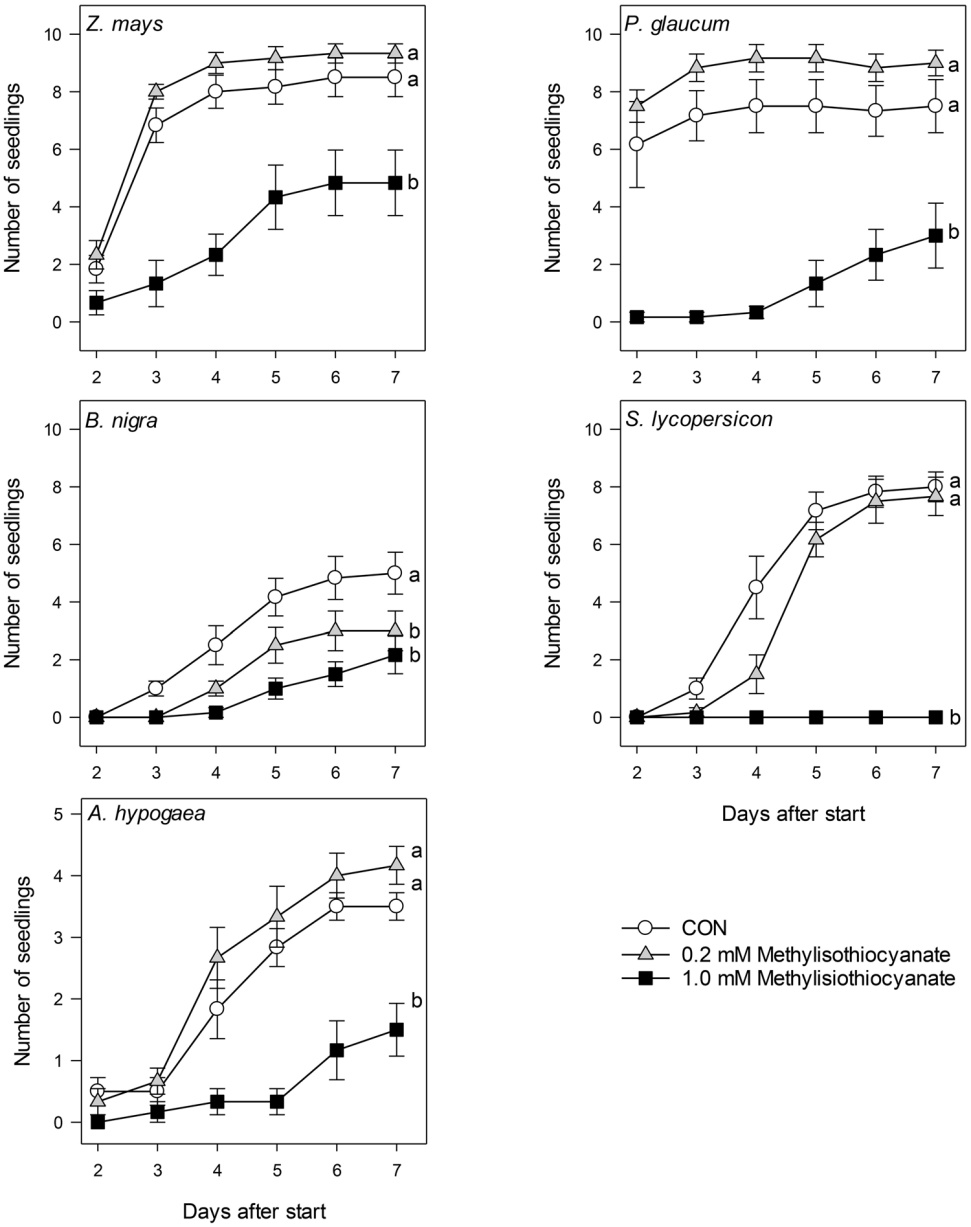
**Numbers of seedlings observed (+SEM) from 2 to 7 days after sowing in pots watered with tap water (Con, open circles), 0.2 mM (gray triangles), or 1.0 mM MeITC (black squares) solution.** Data were analyzed using Kaplan Meier survival analyses. Different letters indicate significant differences in germination after pairwise comparisons between treatments, followed by Holm’s correction for multiple comparisons.

Germination experiments were repeated for *Z. mays*, *P. glaucum*, *B. nigra*, *S. lycopersicon*, and *A. hypogaea* using MeITC concentrations equivalent to those found in the waste water. Water containing 1.0 mM of MeITC reduced or delayed germination in all species tested (**Figures 1F–H** and **5**; Kaplan Meier survival analysis; *Z. mays*, Chi-square: 52.09; *P. glaucum*, Chi-square: 85.87; *B. nigra*, Chi-square: 15.78; *S. lycopersicon* Chi-square: 87.63, and *A. hypogaea* Chi-square: 23,74; d.f. = 2 and *p* < 0.001 for each test). Pairwise comparisons between the different treatments groups within each plant species (McNair et al., 2012) showed that overall the 0.2 mM concentration did not strongly inhibit germination. In three species, *Z. mays, P. glaucum, and A. hypogaea* the low concentration of MeITC showed a mild, yet not significant, stimulatory effect. Similar to the high waste water treatment, the 1.0 mM MeITC solution strongly delayed or even inhibited germination for at least 7 days, thus supporting our hypothesis that MeITC in the high waste water treatment was sufficient to cause the observed effects.

## Discussion

Hanza contains very high levels of MeGSL. Despite earlier calls to determine whether the ‘lengthy preparations of the food successfully detoxify it’ (Booth and Wickens, 1988), to our knowledge this is the first record showing that soaking indeed reduces the high levels of MeGSL in hanza to less than 5% of the original concentration within a week. This indicates that the high levels of glucosinolates are the likely cause of the bitter taste in unprocessed seeds. About 50% of the MeGSL present in the seeds are not retrieved in the waste water or in the sweet hanza at the end of the processing, likely because they are being converted into other compounds. This was corroborated by the observation that the breakdown product MeITC was found in the waste water and its levels followed a similar course in time during the soaking process as MeGSL concentrations in the seeds. Furthermore, we experimentally assessed that the MeITC in the waste water is responsible for the observed allelopathic effect on seed germination in 11 different plant species. Consequently, the MeITC in the waste water reduces its potential use for other activities after the “debittering” process, unless the waste water can be used to control weeds in a sustainable and cost effective manner. Toward this goal, hanza waste water may be used as a natural herbicide. The “Croplife foundation” reports weeding as one of the most taxing and expensive – both physically and economically – labors in crop production in Africa (Gianessi, 2009). Most of this weeding is done by hand and by female farmers. Obtaining economically and environmentally sustainable methods of weed control could significantly impact crop yields in smallholder farms and become part of integrated pest management programs. Of course, negative effects on the crop itself should be avoided. Our data show that the germination of crops and weeds were both affected. However, adult crop plants may be less susceptible; field observations in Niger showed that larger *Sorghum* plants are not negatively affected by watering with hanza waste water (Renate Garvi, personal observations). Moreover, the structurally closely related glucosinolate conversion product allyITC, only caused significant effects on plant growth at concentrations that were at least 10–50 times higher than used in our study (Øverby et al., 2015).

As shown before in *B. senegalensis* from Senegal, the levels of MeGSL were found to differ between sampling times and populations (Gueye et al., 2013b). These studies reported that leaves overall had higher or similar MeGSL levels than the fruits. For unripe fruits, which were extracted as a whole, we found similar results, but in ripe fruits, we showed that the embryo plus the cotyledons, i.e., the hanza, have much higher MeGSL levels than the carpels or the leaves. The MeGSL levels in whole fruits thus are diluted by the extremely low levels in the carpels.

From an ecological perspective, there may be many functions for the high levels of MeGSL in the embryo and the cotyledons, as well as for the low levels of the same compounds in the carpels. Seen the fact that *B. senegalensis* leaves and fruits are used to deter insects that are storage pests (Seck et al., 1993; Gueye et al., 2011), it is likely that the high levels of MeGSL in hanza have evolved to protect their highly valuable seeds from predators. Moreover, it may prevent birds and mammals, the main dispersers of the seeds, to consume the embryo itself. In fact, Tréca and Tamba (1997) report that birds (and mammals to some extent) will consume the fruit. They mostly feed on the outer layers, and later regurgitate or defecate the seeds. Seeds that pass the digestive tract were found to have a higher germination rate than untreated seeds (Tréca and Tamba, 1997). Birds thus are very important for the dispersal of *B. senegalensis*, which may also explain the difference in MeGSL levels between the outer layers of the fruit and the seed. The dispersers would be attracted to the pulp in the sweeter outer layer, while leaving the bitter-tasting seed intact. Moreover, the high level of MeGSL and the MeITC produced when the seed is imbibed may function as allelopathic agents to secure resources in an ecosystem where a single bout of rain may cause a mass germination event, followed by strong competition between and within species. High levels of MeGSL in the seeds may help the *B. sengalensis* propagules to defend their territory against competitors by delaying their germination just long enough to gain a competitive edge (Brown and Morra, 1997; van Dam and Baldwin, 2001). Interestingly, low levels of MeITC appeared to enhance germination in our study. Possibly other species perceive low MeITC levels in the soil as signal for a potential competitor, and respond accordingly by germinating faster.

The allelopathic properties of waste water and MeITC were quite strong, even though we used unsterilized potting soil for the germination tests. In many studies allelopathic effects are analyzed with filter paper as the substrate, which may cause an overestimation of the effectiveness of the compound (Teasdale and Taylorson, 1986). Moreover, we have shown here that the hanza waste water strongly inhibits germination of almost every plant species analyzed so far, particularly the water from the first 3 days of processing. Our test set included plants from several families – Solanaceae, Poaceae, Fabaceae, Malvaceae, and Brassicaceae – both crops and wild plants. We found no clear indications that some families are more susceptible or that crops are more susceptible than weeds. Not surprisingly, the least affected plant species were members of the Brassicaceae (*B. nigra* and *B. juncea*), which themselves contain glucosinolates – albeit different ones – perhaps making them more resistant to glucosinolate breakdown compounds. It would be interesting to assess the effect of the water on the germination of hanza itself. Unfortunately, the viability of dry *B. senegalensis* seeds is very limited and germination rates are low (Daffalla et al., 2011) and thus this was not tested.

Experiments using MeITC at similar concentrations as those found in waste water had similar results as using the waste water. MeITC has been previously reported to inhibit germination of weed seeds and crops (Teasdale and Taylorson, 1986; Brown and Morra, 1997). In addition, the MeITC in the hanza water may also be used to reduce soil pathogens such as pathogenic fungi and nematodes (Brown and Morra, 1997; Matthiessen and Kirkegaard, 2006). As such, hanza water or biomass may be used as a natural and biodegradable alternative to synthetic and persistent soil fumigants such as metam sodium and methylbromide (Matthiessen and Kirkegaard, 2006). More detailed experiments in Niger are needed to show how and when to apply the waste water as weed germination suppressant in various cropping systems without harming the crop or non-target organisms. For example, exposure to MeITC has been shown to be irritating to the eyes and respiratory mucous membrane in humans (Dourson et al., 2010; EPA, 2013). Moreover, constant exposure toxicity tests have shown that MeITC is detrimental to amphibians. Concentrations between 1 and 1000 ppb caused 100% mortality by day 7 in tests with *Xenopus laevis* embryos (Birch and Prahlad, 1986). Concentrations of 248 ppb caused malformations in zebrafish embryos (Haendel et al., 2004). Finally, MeITC may also inhibit the growth of various beneficial soil microorganisms, such as arbuscular mycorrhizal fungi or plant growth promoting rhizobacteria. Thus, as with synthetic pesticides, it is important to first experimentally assess the severity of these potential non-target effects before hanza waste water can be widely exploited as weed suppressant.

In addition to the MeITC, there may be other compounds in the water that could affect germination. Our GC-MS analyses showed the presence of 2,5-dimethyl-1,3,4,-thiadiazole (Supplementary Figure S2) a sulfur containing compound belonging to a class with a wide range of applications as a herbicide and pesticide in agriculture (Hu et al., 2014). Additionally, the relatively high boron levels in the waste water may have phytotoxic effects. Even though direct effects on germination have not been reported, some plant species, such as *V. unguiculata*, show toxicity symptoms at 0.5 mg B per liter irrigation water (see Ayers and Westcot, 1994). Waste water of the first 3 days contains an equivalent of 0.24 mg/L. With time, B may accumulate in the soil when regularly irrigated with water containing high B levels (Camacho-Cristóbal et al., 2008). Additionally, the high amount of starch present in the waste water may create osmotic stress, and consequently prevent germination.

## Conclusion

The processing clearly results in a decrease of glucosinolates in the hanza, thus resulting in a sweet and palatable food source. Nevertheless, the processing also delivers a bitter waste water that is undrinkable and causes allelopathic effects in crops and weeds alike. However, this disadvantage could be proven to be an asset, particularly for small holder farmers, by being used as a natural and cost effective soil fumigant.

## Conflict of Interest Statement

The authors declare that the research was conducted in the absence of any commercial or financial relationships that could be construed as a potential conflict of interest.
