# Supplementary figures and images for: Allelopathic effects of glucosinolate breakdown products in Hanza [Boscia senegalensis (Pers.) Lam.] processing waste water
Source: Front Plant Sci. 2015 Jul 14;6:532. doi: 10.3389/fpls.2015.00532 (PMC4500904; doi:10.3389/fpls.2015.00532)

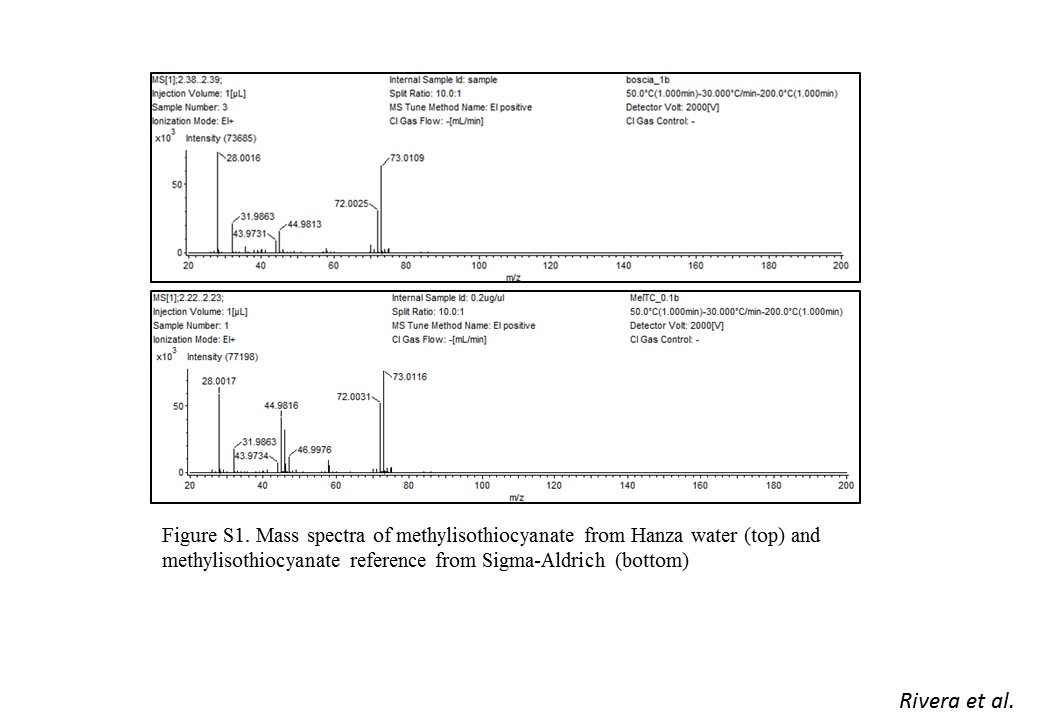

Supplement: Supplementary file 1 [file Image_1.JPEG]

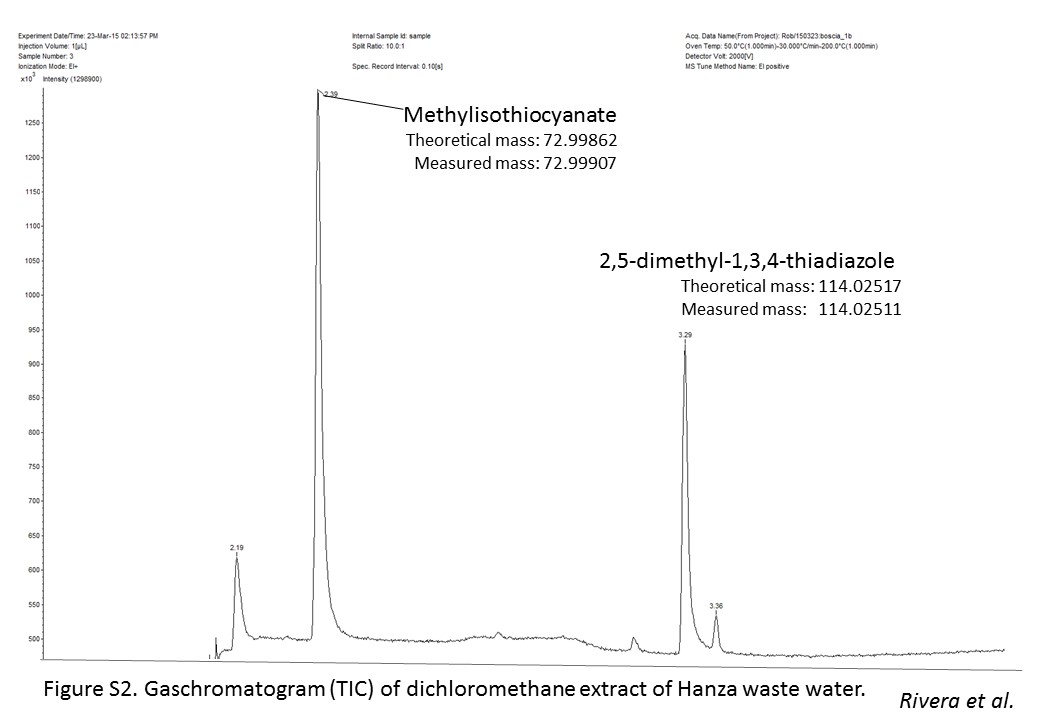

Supplement: Supplementary file 2 [file Image_2.JPEG]
